# One year follow-up and mediation in cognitive behavioral therapy and acceptance and commitment therapy for adult depression

**DOI:** 10.1186/s12888-020-03020-1

**Published:** 2021-01-14

**Authors:** Jacqueline G. L. A-Tjak, Nexhmedin Morina, Maurice Topper, Paul M. G. Emmelkamp

**Affiliations:** 1 Skils, Dr van Deenweg 98, 8025 BJ Zwolle, The Netherlands; 2 Institute of Psychology, University of Münster, Fliednerstr. 21, 48149 Münster, Germany; 3 GGZ-Noord-Holland-Noord, Stationsplein 138, 1703 WC Heerhugowaard, The Netherlands; 4 Department of Clinical Psychology, University of Amsterdam, Nieuwe Achtergracht 129 B, 1018 WS Amsterdam, The Netherlands

**Keywords:** Acceptance and commitment therapy, Cognitive behavior therapy, Depression, RCT, Follow-up, Mediation

## Abstract

**Background:**

Existing therapies for depression are effective, but many patients fail to recover or relapse. To improve care for patients, more research into the effectiveness and working mechanisms of treatments is needed. We examined the long-term efficacy of Cognitive Behavioral Therapy (CBT) and Acceptance and Commitment Therapy (ACT) for Major Depressive Disorder (MDD), testing the hypothesis that CBT outperforms ACT and that both therapies work through their designated mechanisms of change.

**Methods:**

We conducted a randomized controlled trial with 82 patients suffering from MDD. Data were collected before, during and after treatment, and at 12-month follow-up, assessing symptoms of depression, quality of life, dysfunctional attitudes, decentering, and experiential avoidance.

**Results:**

Patients in both conditions reported significant and large reductions of depressive symptoms (*d* = − 1.26 to − 1.60) and improvement in quality of life (*d* = 0.91 to − 1.28) 12 months following treatment. Our findings indicated no significant differences between the two interventions. Dysfunctional attitudes and decentering mediated treatment effects of depressive symptoms in both CBT and ACT, whereas experiential avoidance mediated treatment effects in ACT only.

**Conclusions:**

Our results indicate that CBT is not more effective in treating depression than ACT. Both treatments seem to work through changes in dysfunctional attitudes and decentering, even though the treatments differ substantially. Change in experiential avoidance as an underlying mechanism seems to be an ACT-specific process. Further research is needed to investigate whether ACT and CBT may work differently for different groups of patients with depression.

**Trial registration:**

clinicaltrials.gov; NCT01517503. Registered 25 January 2012 - Retrospectively registered.

## Background

According to the World Health Organization (WHO [1];), more than 300 million people worldwide are affected by depression. Recent estimates by Hoppen and Morina [2] indicate even higher numbers. Several evidence-based treatments are available for adults with depression. Meta-analyses and reviews have shown that different treatments are effective (e.g., [3, 4]). Cognitive Behavioral Therapy (CBT) has been researched the most. Yet, only about 60% of those receiving CBT for depression recover from this condition (e.g., [5]). Relapse constitutes a serious problem in the treatment of depression [6]. Optimizing treatment for patients is an important task for clinicians and researchers. New therapies, such as Acceptance and Commitment Therapy (ACT), have been developed with this purpose. Several studies on ACT for depression have indicated that ACT can successfully treat depression [7–9].

### Mechanisms of change in treatments for depression

Investigating mechanisms of change may help to optimize treatment for patients with depression [10]. One important way to investigate mechanisms of change is mediation, pointing to an intervening variable that may (statistically) explain the association between the dependent (outcome) and independent (treatment) variable [11]. Several potential mediators have been proposed in the literature in relation to depression. Cognitive theory states that depression is caused and maintained by dysfunctional cognitions and maladaptive information processing strategies [12, 13]. According to cognitive theory, depression severity can be reduced by altering the function, content and structure of cognitions and schemas associated with negative affect [14]. Therefore, most of the studies on mediation in the treatment of depression examine cognitive processes, either focusing on changing the content, such as automatic thoughts and dysfunctional attitudes, or on changing the process, such as attributional style, cognitive reactivity, and rumination.

In a review of 31 trials that investigated cognitive processes in cognitive therapy, support was found for the role of cognitive change in content [15]. However, change in content also occurred following interventions that did not target cognitions. Furthermore, the influence of other factors on depression is still largely unknown, as few studies investigated processes other than change in cognitive content, [16].

The therapeutic model of ACT describes six therapeutic processes that together lead to change in psychological flexibility [17]. All processes have a flexible and inflexible counterpart. As such, it offers six possible mediators: experiential avoidance/acceptance, fusion/defusion, present moment (non)awareness, fusion with self-image/flexible perspective taking, (weak) contact with values and (lack of) committed action. Of these processes, experiential avoidance/acceptance has been studied the most. Experiential avoidance refers to the tendency to inhibit or change aversive emotions, thoughts or bodily experiences. Acceptance, its counterpart, is a process of actively choosing to contact private experiences in the service of more behavioral freedom. Changing the content of thoughts is seen as an unnecessary step in ACT, as it is assumed that distancing oneself from thoughts is a sufficient and more productive way to diminish the influence of thoughts on behavior. Distancing is achieved through the process of defusion or decentering. Trials that directly compare mediation variables between different therapies may help to understand whether different interventions are functionally distinct from each other. To our knowledge, only one published study investigated mediation of treatment efficacy when comparing CBT to ACT for depression [18].

### Problems in the research of mediation

Most of the research on mediation in depression has been of a correlational nature, focusing on potential mediators. Although cross-sectional studies on mediation contribute to the mapping of possible mediators, true statistical mediation requires ascertaining the direction of causality. For this, it is important that both the candidate mediator and outcome are assessed at multiple time-points during and after treatment. A process of change can only be considered a mediator when changes in that process precede changes in outcome [11]. In a review of potential mediators reported in current literature about psychotherapy for depression, Lemmens et al. [16] concluded that the field needs to improve the methodology of research, in order to demonstrate the causal relation between change in the mediator and change in depressive symptoms. For this, modern statistical analyses are needed. For instance, traditional mediation models focus on inter-individual differences. Newer mediation models, like Latent Growth Curve Modeling (LGCM), additionally allow intra-individual variation as a part of a mediation process [19].

Lemmens et al. [16] describe six criteria a study needs to meet in order to being able to measure potential mediators of behavior change: 1) the use of an randomized controlled trial design, 2) inclusion of a control group, 3) a sufficient sample size (defined as *n* ≥ 40), 4) examination of multiple potential mediators within one study, 5) the assessment of temporality (three or more assessment points in the treatment phase), and 6) direct experimental manipulation of the mediator. The authors found only 4 out of 35 studies in various forms of psychotherapy for depression that met at least 5 of 6 of the above mentioned criteria for mediation. Of those, only the study by Forman et al. [20] confirmed the original hypotheses, that cognitive techniques facilitated outcome for those receiving CBT, whereas utilization of psychological acceptance strategies facilitated outcome in ACT.

In conclusion, there is a need for methodologically sound mediational research, based on a solid theory of change, capable of establishing the temporal precedence of the mediator relative to outcome measures, and with the use of proper statistical methods.

### The current study

The aims of this paper are twofold. First, to present results on the efficacy 12 months after treatment, of CBT and ACT in patients with MDD. When we started our trial in 2011, findings on the efficacy of CBT for depressive symptoms were already robust [21] whereas studies into the efficacy of ACT were scarce. In fact, CBT was considered a first-line treatment of choice as superiority of CBT over alternative therapies seemed evident among patients with depressive disorders [22]. Hence, we hypothesized CBT to perform better than ACT. Recently, we reported the results of our study, finding no difference in efficacy between ACT and CBT in the treatment of MDD at post-treatment as well as 6 months after treatment [7]. The current paper focuses on the efficacy of ACT and CBT 12 months after treatment. The second aim of the paper was to investigate the extent to which dysfunctional attitudes, decentering and experiential avoidance mediated treatment outcome of depressive symptoms. Experiential avoidance and dysfunctional attitudes have been studied before, mostly in correlational research. We included these processes in our study to investigate their causal role. Furthermore, since according to the ACT model, changing cognitive thought is deemed unnecessary, as distancing would already diminish the influence of cognitions on behavior, we also added decentering in our research. In particular, we had four hypotheses: 1) Treatment gains will be maintained 12 months after termination of treatment for both the CBT and ACT condition on measures of depression and quality of life, 2) CBT will outperform ACT in the treatment of depression at 12 months follow-up, 3) Changes in dysfunctional attitudes, decentering and experiential avoidance will mediate treatment outcome. 4) Treatment-specific processes will mediate treatment outcome within the specified treatment only; for CBT, dysfunctional attitudes mediate treatment outcome, whereas for ACT, decentering and experiential avoidance mediate treatment outcome.

## Methods

### Sample size calculation

An a-priori power calculation in G*Power on the basis of the F-test family, choosing ANOVA (repeated measures within-between interaction) as the statistical test, setting the number of measurements at 4 (pre-treatment, post-treatment, 6 months follow-up, 12 months follow-up), with a correlation of .35 between repeated measures, nonsphericity set at .80, the significance level at .05 (two-sided), a power of .80 and an expected effect of medium size (f = .25) resulted in a required total sample size of 42. Accounting for attrition, we were able to assess a larger number of participants (*n* = 52) at 12 months follow-up.

### Participants

Eligible participants were patients who were referred for treatment to PsyQ, an outpatient treatment facility, by mental health practitioners. MDD had to be the principal diagnosis, as assessed with the Structured Clinical Interview for DSM-IV Axis I Disorders (SCID I [23];). Patients were between 18 and 65 years. The following (comorbid) disorders were excluded: bipolar, psychotic, substance dependence, borderline or anti-social personality and organic brain syndrome. Antidepressant medication was required to be on a stable dose. In accordance with the policy of the Mental Health Institute where the research was conducted, this meant that after using the same dose for at least two weeks, it was considered to be stable enough to enroll in the study and receive psychological treatment. Patients were not allowed to receive other individual psychological treatments than the one they were assigned to in our study. We assessed 601 subjects for eligibility, of whom 99 were randomized and 82 received treatment within our study. Reasons for not being eligible were mostly not meeting inclusion criteria and declining to participate. After randomization, main reasons to not participate in the study were: not showing up for treatment, declining treatment and misunderstandings in the treatment team. Patients were randomly allocated to CBT (*n* = 38) or ACT (*n* = 44). More information about randomization and a CONSORT diagram can be found in A-Tjak et al. [7].

### Therapists

Therapists were clinical psychologists, who had completed at least 100 h of CBT courses and had 2 years of experience in practicing therapy. Two senior therapists were assigned, one to each treatment. All other therapists were randomly assigned to either of the two treatments. Therapists filled out forms in which they reported what kind of interventions were used. These forms were discussed and therapy was monitored during supervision. Eight therapist provided ACT, six provided CBT. Given that the CBT therapists were already experienced in applying CBT and the ACT therapists had no experience in applying ACT, prior to the study (with one exception), the therapists in the ACT condition received 4 days of training, whereas the CBT therapists received only 2 half-days of training. Also, during the first year of their participation in the study, the ACT therapists received twice as much group supervision as CBT therapists. More information on methods can be found in A-Tjak et al. [7].

### Interventions

Treatment in both conditions consisted of a maximum of 20 sessions over 30 weeks. The amount of treatment sessions could be adjusted to the patient’s needs, with a minimum of 9 and a maximum of 20 sessions. In the ACT condition, the mean of treatment sessions was 15.02 with a standard deviation of 5.75. For the CBT condition this was 14.89 and 5.60 respectively, with no significant difference between the two conditions (*p* = .92). CBT was provided in a manner consistent with standard CBT for depression [14] and included both behavioral and cognitive aspects in an integrated fashion [24]. The first eight sessions (phase one) addressed BA and social skill development. The next eight sessions (phase two) addressed cognitive dispute and changing the content of thinking.

ACT was provided through the treatment manual for depression developed for this study by the first author [25], based on Hayes et al. [17], Zettle [26] and Robinson and Strosahl [27]. The manual consists of 16 sessions addressing acceptance, defusion, an observing perspective and present moment awareness in the first eight sessions. The subsequent part of the treatment addressed behavioral change through values clarification and shaping committed action.

In short, we found that at post-treatment, remission rates from depression were 75 and 80% for the ACT and CBT conditions, respectively. Patients in both conditions further reported significant and large reductions of depressive symptoms and improvement on quality of life from pre- to post-treatment as well as at the 6-month follow-up. Our findings indicated no significant differences between the two intervention groups at post-treatment and at 6-month follow-up. More information on pre- vs. post-treatment and 6-month follow-up efficacy related results of the trial can be found elsewhere [7].

### Measures

#### Primary outcomes

Measures used were the Structured Clinical Interview for DSM–IV [23] for depression, the QIDS-SR [28] and the Hamilton Depression Rating Scale [29]. Here, we only report on the results from the QIDS-SR. The 16 items of this scale assess typical depressive symptoms such as sleep disturbance, psychomotor activity and changes in appetite or weight. The QIDS-SR total score ranges from 0 to 27. A score of 0–5 is considered to be within the normal range. In the current study, the Cronbach alpha ranged from 0.68 to 0.87 across assessments.

#### Secondary outcome

The European Health Interview Surveys Quality of Life Scale (EUROHIS) was used to measure quality of life [30]. Scores on this scale can range from 8 to 40, higher scores indicating higher quality of life. The alpha coefficients for this measure in the current study ranged from 0.73 to 0.83 across assessments.

#### Potential mediators/underlying mechanisms

Dysfunctional attitudes were assessed with the Dysfunctional Attitude Scale-revised (DAS-17 [31];). This is a 17-item self-report inventory used to assess excessive and rigid beliefs, hypothesized by Beck [32] to be vulnerability factors for depression. Scores range from 17 to 119, with higher scores indicating greater levels of dysfunctional attitudes. The alpha coefficients for this measure in the current study ranged from .92 to .94 across assessments.

Decentering was assessed with the 11-item Decentering subscale of the Experiences Questionnaire (EQ-D, [33]) a self-report instrument. Scores can range from 13 to 65. The higher the score, the better a person is in distancing himself from his thoughts. The alpha coefficients for this measure in the current study ranged from .73 to .93 across all assessments.

Experiential avoidance was measured with the Acceptance and Action Questionnaire (AAQ-II). Originally, this is a ten-item self-report measure of acceptance or the inflexible counterpart of emotional/experiential avoidance. Recent developments have shown that the seven-item version is probably a good alternative [34], and that version was used in this study. The Dutch version was adapted from Jacobs et al. [35]. The total score ranges from 7 to 49. High scores indicate less experiential avoidance or more acceptance. The alpha coefficients for this measure in the current study ranged from .86 to .95 across all assessments.

Comparing equally effective treatments at the level of mediation helps determine whether interventions are functionally distinct from each other [16]. For this reason, measures of potential mediators for one condition were also administered in the other condition.

### Data collection

Outcome data (QIDS-SR and EUROHIS) were collected pre- and post-treatment, at 6-month and at 12-month follow-up. DAS-17, EQ-D and AAQ-II were assessed pre- and post-treatment. Furthermore, QIDS-SR, DAS-17, EQ-D and AAQ-II assessments also occurred at the 1st, 6th, 11th and 16th session during treatment.

### Data analytic approach

Consistent with the initial report of this trial [7] multilevel analyses were conducted to evaluate and compare the effect of the CBT and ACT interventions on symptoms of depression and quality of life at 12-month follow-up. The level-1 model included the time variable, which captures within-person change over time. In the level-2 model, between-person characteristics such as intervention condition were used to predict the slope estimates representing change in the dependent variables. Maximum likelihood estimation was employed providing unbiased estimates in the case of missing data. The assumption that data were missing at random was evaluated by using binary logistic regression to predict measurement drop-outs. Baseline characteristics did not differ significantly between conditions (all *ps* > .05). Subject-specific random effects (i.e., random intercept and slope) were retained whenever they significantly contributed to the model. Due to the absence of a significant covariance between the intercept and the slope for all measures, we defined a diagonal covariance structure of random effects at level 2.

We estimated a linear trend indicating the direction and rate of change, a quadratic trend indicating a first reversal in the rate of change, and a cubic trend indicating a second reversal in the rate of change (e.g., relapse of symptoms). Contrast coding was used to evaluate the effect of the categorical variable intervention condition [36]. The contrast compared the interventions (ACT coded .5 and CBT coded −.5). Any differences in the rate of change of depression and quality of life between the 6-month and 12-month follow-up assessments were represented by a Cubic Time × Intervention Condition interaction.

Within group effect sizes (Cohen’s d) for each outcome measure were calculated by dividing the difference between the pre-treatment and 12-month follow-up means by the standard deviation of each mean. To correct for dependence among these means [37], we calculated the correlation between the pre-treatment and 12-month follow-up scores. Between-group effect sizes (Cohen’s d) from pre-treatment to 12-month follow-up were calculated by subtracting the means and dividing the result by the pooled standard deviation, adjusting the calculation of the pooled standard deviation with weights for the sample sizes. We used the pooled pre-treatment standard deviation for weighting the differences of the pre-treatment to follow-up means as proposed by Morris [38]. In both calculations the CBT group was treated as the control group.

Structural equation modelling was conducted in Mplus 6.12 [39] to evaluate the effects of the potential mediators dysfunctional attitudes, decentering and experiential avoidance on symptom levels of depression. Mediation was assessed in three steps. First, latent growth curve modelling [40, 41] was conducted to assess change in the mediators during the treatment phase. For each individual mediator, an intercept-only, linear change and quadratic change model was fitted to assess which model best described the shape of change (i.e., no change versus linear change versus curvilinear change) across six time points; pre-treatment, session 1, 6, 11, 16, and post-treatment. Separate models were also fitted for the primary outcome of depressive symptoms (QIDS-SR scores) to obtain estimates of initial levels (i.e., intercept), growth (i.e., slope), and to determine the extent of variation across individuals. The multigroup modeling approach [42] was applied to test whether change in the mediators and outcome was of different magnitude for ACT and CBT. Bayesian Information Criterion (BIC [43];) was used to examine the relative strengths of the candidate models, with smaller values indicating a better fit of the model to the observed data. According to Raftery [44], BIC differences < 2, between 2 and 6, and > 6 indicate respectively, weak, moderate, and strong evidence in favor of the model with the lowest values. The absolute model to data fit for each of the models was evaluated using the Root Mean Square Error of Approximation (RMSEA) and the Comparative Fit Index (CFI).^1^ For interpretation of these model fit indices we followed Hu and Bentler [47]. That is, RMSEA values less than .06 were taken as an indication of a good model fit whereas values between .06 and .10 were taken as an indication of an acceptable model fit. For the CFI, values of .90 or higher were taken as an indication of acceptable model fit, and values of .95 or higher were taken as an indication of good model fit.

In a second step, the best fitting growth curve model for each mediator was combined with the best fitting growth curve model of the outcome in a series of parallel process models. These models allowed us to investigate whether change in each mediator was associated with change in the outcome (i.e., a significant mediator slope to outcome slope regression path) supporting the hypothesis of common developmental trends among mediators and outcome.

As we have discussed in the introduction, mediation requires that change in the mediator occurs before change in the outcome variable [16]. One criticism of the parallel process model of mediation is that it merely assesses mediation effects for contemporaneous change as opposed to mediation effects for longitudinal change in which prior change in the mediator is related to subsequent levels in the outcome variable [48]. In a third step, we investigated longitudinal mediation by applying latent difference score models [49]. In these models, successive latent difference scores for each of the mediators were derived by fixing two paths at 1, the path from the starting point to the end point of an interval (e.g., session 1 to session 6) and the path from the latent difference of that interval to the last time point from which that difference score was derived (e.g., latent difference of session 1 to 6 to the session 6 time point). The residuals of the mediators were set equal to 0. Because of these constraints, latent difference scores for each of the five intervals were obtained that represent dynamic change in terms of the difference between the intervals. The mean of the observed depressive symptom scores was estimated along with the means of the latent difference scores for the mediators. The latent difference scores were used to predict symptom levels of depression at the end of that interval (e.g., latent difference of mediator from session 1 to 6 predicting QIDS-SR scores at session 6) while controlling for earlier depressive symptom levels through the estimation of autoregressive pathways. The multigroup modeling approach was applied to test whether mediation effects were treatment specific as formulated in hypothesis 4 (ACT: decentering and experiential avoidance, CBT: dysfunctional attitudes).

Response rates at the different assessment points varied. All participants filled out the pre-treatment assessment, whereas response rates at the 16th session, at 6-month follow-up and at 12-month follow-up were lowest with 61.0, 70.7, and 63.4% completion, respectively. Response rates at the remaining time points ranged from 80.5 to 98.8%.

For all models the maximum likelihood missing data procedure was used. This maximum likelihood procedure uses all available data from each participant and assumes that data are missing at random. A Little MCAR’s test was performed to determine the pattern of missingness. The results of this test indicated that missing values were completely at random, χ2(41) = 46,58, *p* = .25.

## Results

### Descriptive statistics

In the ACT condition, we assessed 25 comorbid disorders, mostly anxiety disorders and dysthymia. The mean number of comorbid disorders was .57. In the CBT condition we assessed 24 comorbid disorders, also mostly anxiety disorders and dysthymia, with a mean of .63. In both conditions 5 participants had comorbid dysthymia. Personality disorders were not assessed, except for borderline and anti-social personality disorder. In the ACT condition 19 participants and in the CBT condition 17 participants, had been treated for depression before. We found no significant differences between the two conditions, with exception of the waiting time between the pre-treatment assessment and the first treatment session, *t* (56.77) = − 4.15. Participants in the ACT condition (*M* = 3.70, *SD* = 2.60) had a shorter waiting time (in weeks) compared to participants in the CBT intervention (*M* = 7.18, *SD* = 4.57, *p* <.001). We therefore entered this variable as a covariate in the main analyses. There were no significant differences between conditions at the pre-treatment assessment (*ps* = .12 to .97) on any of the other measures. More information can be found in A-Tjak et al. [7].

### One year outcome

Depressive symptom scores (QIDS-SR) and scores of quality of life (EUROHIS) are presented in Table 1. Results from post-treatment and 6-month follow-up have already been reported in our previous article [7]. We present them here for the convenience of the reader. The results of multilevel analyses can be found in Table 2. Patients reported large reductions in depressive symptoms from pre-treatment to 12-month follow-up. There were no differences in the rate of change between ACT and CBT from 6-month follow-up to 12-month follow-up, as indicated by a non-significant Cubic Time × Condition interaction. Patients also reported large increases in quality of life from pre-treatment to 12-month follow-up. The Cubic Time × Condition interaction was not significant, indicating no differences between ACT and CBT in the rate of change in quality of life from 6-month follow-up to 12 month follow-up.

**Table 1:** Corrected mixed-regression based estimated means, standard errors (in parentheses) and within- and between-group effect sizes (Cohen’s *d*) at 12 m follow-up

Measure	Condition	Pre *M* (*SE*)	Post *M* (*SE*)	6 m FU *M* (*SE*)	12 m FU M (*SE*)	Within-group *d*	Between-group *d*^a^
QIDS-SR	ACT	14.96 (0.92)	7.89 (0.88)	7.24 (0.84)	7.52 (0.87)	−1.26*	−0.25
	CBT	14.61 (0.99)	6.31 (0.96)	7.15 (0.94)	5.66 (0.94)	−1.60*	
EUROHIS	ACT	20.16 (0.93)	24.95 (0.85)	24.33 (0.79)	25.24 (0.85)	0.91*	0.48
	CBT	20.79 (1.00)	26.10 (0.94)	26.46 (0.87)	27.38 (0.92)	1.28*	

*Note. ACT* Acceptance and Commitment Therapy, *CBT* Cognitive Behavioral Therapy, *EUROHIS* European Health Interview Surveys Quality of Life Scale, *QIDS-SR* Quick Inventory for Depressive Symptomatology Self-Rated

^a^ Cohen’s *d* pre- treatment to 1-year follow-up effect size

* *p* < .001

**Table 2:** Multilevel regression analyses for ACT and CBT on depressive symptoms (QIDS-SR) and quality of life (EUROHIS)

Measure	*F*	*df*	*b*	*SE*	*p*
**QIDS-SR**					
linear time	53.90	84.65	−2.02	.28	<.001
quadratic time	68.02	144.73	1.75	.21	<.001
cubic time	19.83	138.96	.35	.08	<.001
waiting time	1.47	81.35	.16	.13	.23
contrast	1.70	106.98	1.49	1.14	.20
Linear time * Condition	.02	84.56	.08	.55	.88
Quadratic time * Condition	.18	144.59	.18	.42	.67
Cubic time * Condition	2.67	139.03	−.26	.16	.11
**EUROHIS**					
lineair time	30.35	71.10	1.40	.25	<.001
quadratic time	29.89	118.47	−1.02	.19	<.001
cubic time	12.97	111.40	−.25	.07	<.001
waiting_time	2.54	80.72	−.21	.13	.11
contrast	1.16	101.51	−1.23	1.14	.28
Lineair time * Condition	1.83	71.04	−.67	.51	.18
Quadratic time * Condition	.03	118.34	.07	.37	.86
Cubic time * Condition	.24	111.45	−.07	.14	.63

*Note. ACT* Acceptance and Commitment Therapy, *CBT* Cognitive Behavioral Therapy, *EUROHIS* European Health Interview Surveys Quality of Life Scale, *QIDS-SR* Quick Inventory for Depressive Symptomatology Self-Rated. Waiting time is the time between pretreatment assessment and first treatment session, in weeks. Condition refers to treatment group (CBT or ACT)

### Mediation

Depressive symptoms, dysfunctional attitudes, decentering and experiential avoidance scores at pre-treatment, during treatment and post-treatment are presented in Table 3. Overall model fit information for the models that were fitted are presented in Table 4.

**Table 3:** Means (SD) of depressive symptoms (QIDS-SR), dysfunctional attitudes (DAS-17), decentering (EQ-D), and experiential avoidance (AAQ-II) from pre-treatment to post-treatment

	Condition	Pre- Treatment (*N* = 82)	Session 1 (*N* = 77)	Session 6 (*N* = 58)	Session 11 (*N* = 49)	Session 16 (*N* = 30)	Post- Treatment (*N* = 67)
QIDS-SR	ACT	14.96 (4.15)	15.06 (4.63)	11.76 (5.08)	11.21 (6.16)	8.84 (5.84)	8.10 (6.54)
	CBT	14.61 (4.55)	13.57 (4.71)	11.24 (5.32)	9.68 (5.66)	8.23 (4.92)	6.34 (5.28)
DAS-17	ACT	63.36 (17.80)	62.15 (19.83)	61.03 (17.89)	59.51 (21.54)	52.42 (16.40)	50.03 (17.42)
	CBT	60.55 (19.95)	59.67 (22.91)	54.33 (18.94)	55.91 (17.23)	51.01 (16.12)	46.56 (16.91)
EQ-D	ACT	34.96 (5.41)	33.91 (6.65)	33.06 (6.28)	35.58 (7.40)	37.63 (7.52)	37.58 (7.57)
	CBT	34.45 (5.61)	33.89 (6.95)	32.08 (6.97)	33.78 (5.29)	36.04 (7.29)	37.13 (5.28)
AAQ-II	ACT	23.77 (6.95)	23.36 (7.70)	24.96 (7.80)	27.92 (9.71)	30.20 (10.60)	30.77 (9.57)
	CBT	23.71 (7.61)	26.52 (8.92)	27.71 (8.89)	25.71 (8.92)	30.17 (8.86)	30.97 (8.70)

*Note. AAQ-II* Acceptance and Action Questionnaire-II, *ACT* Acceptance and Commitment Therapy, *CBT* Cognitive Behavioral Therapy, *DAS-17* Dysfunctional Attitude Scale-revised, *EQ-D* Decentering subscale of the Experiences Questionnaire, *QIDS-SR* Quick Inventory for Depressive Symptomatology

**Table 4:** Goodness-of-fit indices for the univariate latent growth curve models, the parallel process models and the latent difference score models

Model	X^2^ (df)	RMSEA	CFI	BIC
**QIDS-SR**				
Intercept-only	234.56 (19)***	0.37	0.16	2451.88
Linear change	17.03 (11)	0.08	0.98	2244.37
Curvilinear change	14.59 (7)*	0.12	0.97	2246.94
Multigroup LGCM ≠^a^	30.01 (24)	0.08	0.98	2241.28
Multigroup LGCM =^b^	30.54 (25)	0.07	0.98	2240.56
**DAS-17**				
Intercept-only	102.37 (19)***	0.23	0.72	2826.02
Linear change^c^	29.01 (16)*	0.10	0.96	2756.41
Curvilinear change	17.25 (12)	0.07	0.98	2749.66
Multigroup LGCM ≠^a^	35.27 (27)	0.09	0.97	2741.72
Multigroup LGCM =^b^	35.31 (29)	0.07	0.98	2739.26
Parallel process model^d^	102.02 (55)***	0.10	0.93	4956.86
Latent difference score model	55.43 (35)*	0.08	0.97	4935.32
Latent difference score model multigroup ≠^e^	236.53 (70)***	0.24	0.79	4946.48
Latent difference score model multigroup =^f^	241.21 (75)***	0.23	0.79	4944.90
**EQ_D**				
Intercept-only	109.26 (19)***	0.24	0.59	2556.74
Linear change	45.46 (11)***	0.20	0.84	2502.95
Curvilinear change^g^	18.11 (9)*	0.11	0.96	2478.11
Multigroup LGCM ≠^a^	60.20 (24)***	0.19	0.86	2480.85
Multigroup LGCM =^b^	60.30 (26)***	0.18	0.86	2478.44
Parallel process model	80.23 (50)***	0.09	0.95	4667.48
Latent difference score model	78.39 (35)***	0.12	0.92	4684.43
Latent difference score model multigroup ≠	178.44 (70)***	0.19	0.83	4665.22
Latent difference score model multigroup =	186.54 (75)***	0.19	0.83	4667.06
**AAQ-II**				
Intercept-only	91.46 (19)***	0.22	0.63	2799.96
Linear change	12.08 (11)	0.04	0.99	2730.59
Curvilinear change	7.52 (7)	0.03	0.99	2731.05
Multigroup LGCM ≠^a^	42.77 (24)*	0.14	0.92	2724.58
Multigroup LGCM =^b^	42.78 (25)*	0.13	0.92	2723.33
Parallel process model^h^	67.66 (54)	0.06	0.98	4892.43
Latent difference score model	93.65 (35)***	0.14	0.90	4942.23
Latent difference score model multigroup ≠	172.53 (70)***	0.19	0.84	4917.40
Latent difference score model multigroup =	191.90 (75)***	0.20	0.81	4930.50

*Note. QIDS-SR* Quick Inventory for Depressive Symptomatology Self-Rated, *DAS-17* Dysfunctional Attitude Scale-revised, *EQ-D* Decentering subscale of the Experiences Questionnaire, *AAQ-II* Acceptance and Action Questionnaire-II, *RMSEA* root mean square error of approximation, *CFI* Comparative Fit Index, *BIC* Bayesian Information Criterion, *LGCM* latent growth curve model. * = *p* < .05, *** = *p* < .001

^a^In this model a multigroup approach was applied to the best fitting univariate LGCM while freely estimating the slope factor means

^b^In this model a multigroup approach was applied to the best fitting univariate LGCM while an equality constraint was imposed on the slope factor means

^c^correlated measurement residuals among adjacent time points were not included for these linear and curvilinear models as this provided a superior fit

^d^the curvilinear slope in DAS-17 scores was removed from this model, as a negative residual variance was obtained for this parameter

^e^In this model a multigroup approach was applied to the latent difference score model while freely estimating the regression pathway linking the latent difference score of the mediator to QIDS-SR scores at the end of the interval

^f^In this model a multigroup approach was applied to the latent difference score model while an equality constraint was imposed on the regression pathway linking the latent difference score of the mediator to QIDS-SR scores at the end of the interval

^g^the correlation between pre-treatment and session 1 scores was negative in this model and was constrained to 0

^h^ the curvilinear slope in AAQ-II scores was removed from this model, as a negative residual variance was obtained for this parameter

#### Depressive symptoms

Model fitting procedures for the univariate LGCMs indicated a poor fit for the intercept-only model and a good fit for the linear model. A comparison of the BIC values suggests that the linear change model provided the best fit. This indicates that the assumption of linear change in depressive symptoms over time is preferred over the assumption of no change and curvilinear change. Parameter estimates of the linear change model indicated a significant linear decrease, *M* = − 1.58, *p* < .001, in depressive symptoms at the group level. The variances of the intercept, 15.79, and the slope, 1.02, were significant, *p* < .01, showing significant individual differences of the initial depressive symptom scores and the rate of change over time. Using a multigroup approach on the linear model, we tested whether the trajectory in the group receiving ACT differed from the trajectory in the group receiving CBT. Inspection of the BIC values indicated that the fit of the model in which the slope factor means were allowed to be estimated differently in the two groups was inferior to the fit of the model in which the slope factor means were constrained to be equal. This implies that the depressive symptom linear slopes were not significantly different from one another.

#### Dysfunctional attitudes

Model fitting procedures for the univariate LGCMs indicated a poor fit for the intercept-only model and an acceptable to good fit for the linear and curvilinear model. A comparison of the BIC values indicates that the quadratic change model provided the best fit. This indicates that the assumption of curvilinear change in dysfunctional attitude scores over time is preferred over the assumption of no change and linear change. Parameter estimates of the curvilinear change model indicated a significant linear decline, *M* = − 1.35, *p* < .001, in dysfunctional attitudes at the group level. The mean of the quadratic slope was −.26, *p* = .045, suggesting an acceleration in the rate of decrease over time. Variances of the intercept, 286.32, and linear slope, 1.22, were both significant, *p* < .001, showing significant between-person variance of the initial dysfunctional attitudes scores and the rate of change over time. The variance of the curvilinear slope was nonsignificant, .25, *p* = .34, suggesting no between-person variability in the reversal of the rate of change. Using a multigroup approach on the curvilinear model, we tested whether the trajectory in the group receiving ACT differed from the trajectory in the group receiving CBT. Inspection of the BIC values indicated that the fit of the model in which the slope factor means were allowed to be estimated differently in the two groups was inferior to the fit of the model in which the slope factor means were constrained to be equal. This implies that the dysfunctional attitudes linear and quadratic slope were not significantly different from one another.

In step 2, the relations among change in dysfunctional attitudes and depressive symptoms were assessed in a parallel process model. Providing support for common developmental trends in dysfunctional attitudes and depressive symptoms, the dysfunctional attitudes slope was significantly associated with the slope in depressive symptoms, as indexed by a regression coefficient of .28, *p* < .01. In addition, the intercepts of dysfunctional attitudes and depressive symptoms were significantly associated, .09, *p* = < .01.

**Fig. 1:**
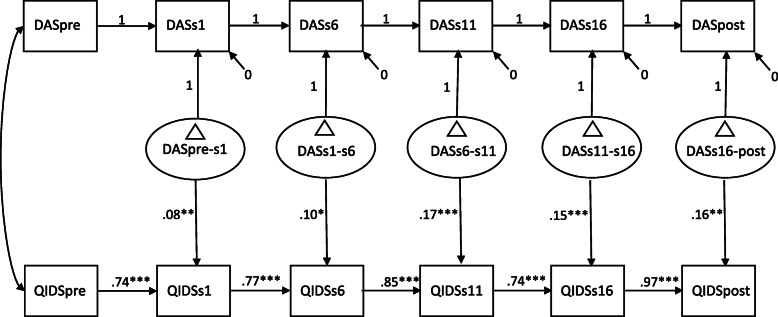
Latent difference score model with dysfunctional attitudes as the mediator and depressive symptoms as outcome. Path coefficients (unstandardized) are aggregated across conditions (ACT, CBT) as this model resulted in the best fit. DAS = DAS-17 scores, QIDS = QIDS-SR scores, pre = pre-treatment, s = session number during treatment, post = post-treatment, ∆ = latent difference score. *** *p* < .001, **p < .01, *p <.05

In step 3, a latent difference score model was fitted. See Fig. 1 for parameter estimates of this model. For each interval, depressive symptom levels at an earlier time point significantly predicted depressive symptom levels at a later time point. Above and beyond these autoregressive effects, each of the dysfunctional attitudes difference score factors significantly predicted symptom levels of depression at the end of the interval. This result indicates that prior change in dysfunctional attitudes was related to subsequent symptom levels of depression for each of the intervals. A multigroup model in which the difference score factors predicting levels of depression were allowed to be estimated differently for ACT and CBT also showed that each of the dysfunctional difference score factors significantly predicted symptom levels of depression at the end of the interval. This model resulted in an inferior fit, relative to a model in which the regression pathways were constrained to be equal across treatment conditions (BIC difference of 1.58 points). Although the fit of both multigroup models was not optimal, this result indicates that, in contrast to what we predicted, the assumption of a non-specific treatment effect of change in dysfunctional attitudes on depressive symptom levels is preferred over the assumption of treatment-specific effects.

#### Decentering

Model fitting procedures for the univariate LGCMs indicated a poor fit for the intercept-only model and the linear change model. The quadratic change model had a considerably better fit, indicating that the assumption of curvilinear change in decentering over time is preferred over the assumption of no change and linear change. Parameter estimates of the curvilinear change model indicated a significant linear increase, *M* = .30, *p* < .01, in decentering at the group level. The mean of the quadratic slope was .25, *p* < .001, suggesting an acceleration in the rate of increase over time. Variances of the intercept, 24.91, linear slope, .38, and quadratic slope, .14, were significant, *p* < .05, showing significant between-person variance of the initial decentering scores, the rate of change over time and the reversal in the rate of change. Using a multigroup approach on the curvilinear model, we tested whether the trajectory in the group receiving ACT differed from the trajectory in the group receiving CBT. Inspection of the BIC values indicated that the fit of the model in which the slope factor means were allowed to be estimated differently in the two groups was inferior to the fit of the model in which the slope factor means were constrained to be equal. This implies that the decentering linear and quadratic slope were not significantly different from one another.

In step 2, the relations among change in decentering and depressive symptoms were assessed in a parallel process model. Providing support for common developmental trends in decentering and depressive symptoms, the decentering slope was significantly associated with the slope in depressive symptoms, as indexed by a regression coefficient of −.71, *p* < .01. In addition, the intercepts of decentering and depressive symptoms were significantly associated, as indexed by a regression coefficient of −.37, *p* = < .01.

**Fig. 2:**
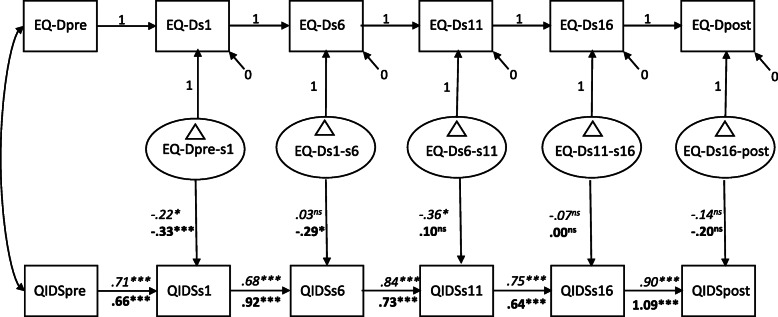
Latent difference score model with decentering as the mediator and depressive symptoms as outcome. Path coefficients (unstandardized) are estimated separately for ACT (italics) and CBT (boldfaced) as this model resulted in a superior fit to a model with equality constraints on the difference score to outcome path coefficients. EQ-D = EQ-D scores, QIDS = QIDS-SR scores, pre = pre-treatment, s = session number during treatment, post = post-treatment, ∆ = latent difference score. *** *p* < .001, **p < .01, *p <.05, ns = non-significant

In step 3, a latent difference score model was fitted. See Fig. 2 for parameter estimates of this model. Above and beyond the significant autoregressive effects, the decentering difference score factors for the first and third interval significantly predicted symptom levels of depression at the end of these intervals. Across treatment groups, this result indicates that changes in decentering from pre-treatment to session 6, and from session 6 to 11 were related to subsequent symptom levels of depression, whereas no evidence for such an effect was found for the remaining intervals. However, a multigroup model in which the difference score factors predicting levels of depression were allowed to be estimated differently for ACT and CBT resulted in a slightly superior fit, relative to a model in which the regression pathways were constrained to be equal across treatment groups (BIC difference of 1.84 points). Although the fit of both multigroup models was not optimal, inspection of the pathways for ACT shows that changes in decentering from pre-treatment to session 1, and from session 6 to 11 significantly predicted subsequent symptom levels of depressive symptoms. For CBT, changes in decentering from pre-treatment to session 1, and from session 1 to 6 significantly predicted subsequent symptom levels of depression. Given the mediational effects of decentering in both conditions, the superior fit of the treatment-specific model potentially results from differences in temporality of these effects across conditions. In contrast to what we expected, these results indicate a non-specific treatment effect of change in decentering on symptom levels of depression.

#### Experiential avoidance

Model fitting procedures for the univariate LGCMs demonstrated a poor fit for the intercept-only model and a good fit for the linear and curvilinear model. Negative residual variances were obtained in the curvilinear model, suggesting that the assumption of curvilinear growth may not be reasonable for the observed data. This indicates that the assumption of linear change in experiential avoidance scores over time is preferred over the assumption of no change and quadratic change. Parameter estimates of the linear change model indicated a significant linear decrease, *M* = 1.38, *p* < .001, in experiential avoidance at the group level. The variance of the intercept was significant, 23.67, *p* < .001, whereas the variance of the slope was nonsignificant, 1.38, *p* = .06, indicating significant individual differences of the initial experiential avoidance scores but no individual differences in the rate of change. Using a multigroup approach on the linear model, we tested whether the trajectory in the group receiving ACT differed from the trajectory in the group receiving CBT. Inspection of the BIC values indicated that the fit of the model in which the slope factor means were allowed to be estimated differently in the two groups was inferior to the fit of the model in which the slope factor means were constrained to be equal. This implies that the experiential avoidance linear slopes were not significantly different from one another.

In step 2, the relations among change in experiential avoidance and depressive symptoms were assessed in a parallel process model. Providing support for common developmental trends in experiential avoidance and depressive symptoms, the experiential avoidance slope was significantly associated with the slope in depressive symptoms, as indexed by a regression coefficient of −.92, *p* < .05. In addition, the intercepts of experiential avoidance and depressive symptoms were significantly associated, as indexed by a regression coefficient of −.31, *p* = < .001.

**Fig. 3:**
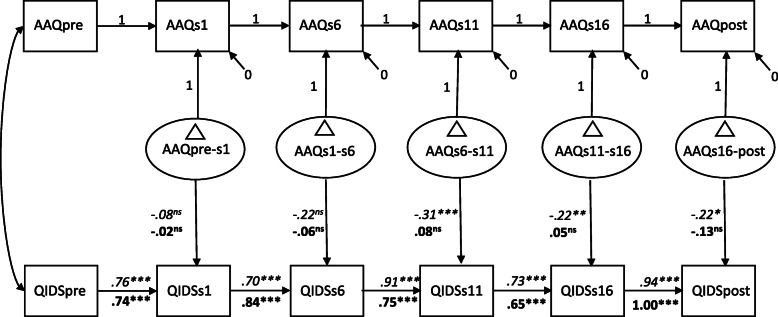
Latent difference score model with experiential avoidance as the mediator and depressive symptoms as outcome. Path coefficients (unstandardized) are estimated separately for ACT (italics) and CBT (boldfaced) as this model resulted in a superior fit to a model with equality constraints on the difference score to outcome path coefficients. AAQ = AAQ-II scores, QIDS = QIDS-SR scores, pre = pre-treatment, s = session number during treatment, post = post-treatment, ∆ = latent difference score. *** *p* < .001, **p < .01, *p <.05, ns = non-significant

In step 3, a latent difference score model was fitted. See Fig. 3 for parameter estimates of this model. Above and beyond the significant autoregressive effects, the experiential avoidance difference score factors for the last interval significantly predicted symptom levels of depression at the end of these intervals. Across treatment groups, this result indicates that changes in experiential avoidance from session 16 to post-treatment were related to subsequent symptom levels of depression, whereas no evidence for such an effect was found for the remaining intervals. However, a multigroup model in which the difference score factors predicting levels of depression were allowed to be estimated differently for ACT and CBT resulted in a superior fit, relative to a model in which the regression pathways were constrained to be equal across treatment groups (BIC difference of 13.10 points). Although the fit of both multigroup models was not optimal, inspection of the pathways for ACT shows that changes in experiential avoidance for all of the intervals, except the first and second interval (pre-treatment to session 1, and session 1 to 6), significantly predicted subsequent symptom levels of depressive symptoms. For CBT, changes in experiential avoidance for all of the intervals did not significantly predict subsequent symptom levels of depression. This result is in line with the expectations, and indicates a specific treatment effect of change in experiential avoidance on depressive symptoms levels for ACT.

## Discussion

This study aimed to examine the 12 month follow-up efficacy and working mechanisms of ACT and CBT among patients with MDD in an outpatient facility. We found large pre-treatment to 12-month follow-up effect sizes for both ACT and CBT (*d* = − 1.26 and *d* = − 1.60 on depressive symptoms and *d* = 0.91and *d* = 1.28 on quality of life). We found no evidence that these changes were larger for CBT than for ACT. Neither did we find significant differences between the CBT and ACT condition in the changes from 6-month follow-up to 12-month follow-up. Taken together, the findings support our first hypothesis that treatment gains were maintained 12 months after termination of both treatments on measures of depression and quality of life. We found, however, no support for our second hypothesis that CBT outperformed ACT in the treatment of depression at 12-month follow-up. The findings are in line with recent meta-analyses on the efficacy of ACT, finding no support for a differential effect of ACT compared to CBT [50, 51].

Changes in dysfunctional attitudes, decentering and experiential avoidance mediated treatment outcomes, as specified in our third hypothesis. We found partial support for our fourth hypothesis that CBT and ACT work through distinct mediators for each treatment. Experiential avoidance mediated outcome in the ACT condition only. Note, however, that the latent growth curve models showed a similar decrease of experiential avoidance for CBT. Yet, the intra- and interindividual changes in this condition could not be systematically linked to subsequent levels of depressive symptoms, as indicated by the non-significant pathways in the latent difference score models. For ACT, the results indicated that intra- and interindividual changes from session 7 onwards predicted subsequent levels of depression pointing to experiential avoidance as a specific underlying mechanism, that differentiates ACT from CBT.

Treatment-specific processes did not mediate outcomes within the specified treatment only, since dysfunctional attitudes and decentering mediated depressive symptom levels for both ACT and CBT. This suggests that ACT and CBT may work for some part through the same underlying cognitive mechanisms. Whereas some of our findings are in line with an earlier study on mediation in CBT and ACT, carried out by Forman et al. [52], other findings clearly diverge from their study. Results of both studies indicate that changes in dysfunctional thinking and defusion mediated both conditions. Both studies found that in the ACT condition, outcome was facilitated by psychological acceptance strategies. Unlike our study, Forman et al. [52] found that the CBT condition outcome was facilitated to a larger extent by cognitive change strategies. The similarities in mediational results are noticeable, given that the sample used in Forman et al. included not only patients with depressive symptoms, but also with anxiety. Some authors underscore the similarities between anxiety and mood disorders and emphasize that a focus on underlying mechanisms of both kinds of disorders is more fruitful than different treatments for different emotional disorders (e.g., [53]).

### The role of dysfunctional attitudes as an underlying mechanism

Our finding that dysfunctional attitudes do not specifically mediate effects of CBT might indicate that change in dysfunctional attitudes is not an underlying mechanism uniquely related to CBT. Cognitive change can be the result of interventions not designed to target cognitive content [15]. Recently, Cristea et al. [54] carried out a meta-analysis that showed that the effects on dysfunctional thinking did not differ significantly between CBT and other psychotherapies or antidepressant medication. An exception, however, resulted when the comparison was restricted to the DAS. The studies included in the meta-analysis by Cristea et al. [54] did not assess changes in the DAS during treatment and according to the authors, the quality of most studies was subpar. Our results, as well as the findings of Forman et al. [52], correspond with their conclusion that a change in dysfunctional beliefs is not a process unique or specific to CBT.

An important factor to consider in understanding the role of mediators is the coordination of applying techniques that are supposed to influence therapeutic processes and measuring change in those processes [55]. In the ACT protocol used in our study, cognitive change interventions were applied during most of the treatment sessions, whereas in the CBT protocol cognitive interventions started in the second half of treatment. This seems to fit with the acceleration in the rate of decrease over time we found. In the ACT condition, explicit defusion skills were administered before session 11, which possibly kept influencing outcome throughout treatment. This effect may have been boosted up in the second half of treatment, when patients were encouraged to use defusion as a strategy to overcome thoughts as barriers for committed action. For the CBT condition, it seems that BA interventions already resulted in cognitive change, and this effect became more prominent during the cognitive phase of treatment. Taken together, our findings indicate that a change in dysfunctional attitudes can be accomplished through different pathways.

Since ACT does not deliberately target change of cognitive content, it may be somewhat unexpected that dysfunctional attitudes were changed in the ACT condition. However, the mediators evaluated in this study may affect each other in a sequential fashion. For example, distancing oneself from one’s own thoughts may offer room for different perspectives and thereby enable the process of change in cognitive content [56]. On the other hand, the availability of more helpful cognitions might help a person gain more distance and acceptance of unhelpful cognitions. These chain reaction effects, referred to as sequential mediation, could not be tested in the current study due to inadequate power leading to convergence errors of the structural equation models. Future studies that are adequately powered may shed further light on the directionality of these effects. Frequent assessment of both mediators and outcome will be crucial for these studies, as sequential mediation effects can only be demonstrated through repeated assessment during treatment.

### The role of decentering as an underlying mechanism

Several studies found a negative relationship between decentering and depression [33, 57–60]. In our study, we found that for participants receiving ACT changes in decentering from pre-treatment to session 1, and from session 6 to 11 significantly predicted subsequent changes in depressive symptoms. For participants receiving CBT, changes in decentering from pre-treatment to session 1, and from session 1 to 6 significantly predicted subsequent changes in depression. It is not uncommon to find differences in the timing of mediation effects [55].

In the CBT condition, decentering seemed to have been triggered during the behavioral phase of treatment. Perhaps, focusing on behavior while no attention is paid to the content of thoughts loosens the relationship between thought and thinker [61]. Later in the treatment, decentering no longer exerted influence on levels of depression anymore. Perhaps this was because in the second half of the treatment the focus was on changing the content of thoughts. This would mean that changing the content of thoughts exerts a different kind of influence than taking a decentered perspective. In the ACT condition, we would expect mindfulness skills to establish decentering. These skills were strengthened during the first phase of treatment. It is possible that these skills started to exert their effects on depressive symptom levels, only when participants reached a certain level of skillfulness, after session 5. After session 11 other unexamined factors may have become more important, such as committed action.

Finding a mediational effect of decentering in both treatment conditions may indicate that taking a distance of cognitive content may be important both in CBT and ACT. This was a premise in the research conducted by Zettle and Hayes [62] and Zettle and Rains [63]. We investigated the role of decentering as a possible mediator, hypothesizing that decentering greatly overlapped with the concept of defusion. Both decentering and defusion focus on distancing from negative thought content, and from other mental events [33]. However, it has been argued that decentering is different from the concept of defusion [64]. Recently, a measure for cognitive defusion, the Cognitive Fusion Questionnaire was developed [65] that might prove useful in future research.

### The role of experiential avoidance as an underlying mechanism

A large body of research supports the mediating role of experiential avoidance and acceptance in psychopathology [66]. Several studies found a relationship between experiential avoidance and depression (e.g., [67, 68]). Experiential avoidance can be both a cause and a consequence of depressive symptoms. Changes in experiential avoidance (more acceptance) lead to less depressive symptoms and vice versa [69]. A decrease in experiential avoidance predicted flourishing after an ACT self-help intervention for adults with depressive symptoms [70]. However, it must be noted that most mediational analyses did not meet the criteria formulated by Lemmens et al. [16].

Since we found experiential avoidance mediating in the ACT and not the CBT condition, reducing levels of experiential avoidance of inner experiences may be an underlying mechanism specific to ACT. In ACT, enhancing acceptance is a specific target of treatment and patients are actively encouraged to use acceptance skills for behavioral change. In our study, acceptance interventions were offered from very early on in the ACT treatment. The other five processes (defusion, present moment awareness, flexible perspective taking, contact with values and committed action) are used to further build this skill. Accordingly, experiential avoidance mediated outcome from session 6 to the end of treatment, showing linear change.

We did not have enough power to study the interactions between the three potential mediators. Mediators can influence each other in a sequential way, but also in a circular way [71]. Furthermore, it is possible that other variables cause the change both in the outcome and in the assumed mediator [72]. Additionally, some mediators have to work in concert to bring about change. Bardeen and Fergus [73] found support for the notion that high levels of cognitive fusion and experiential avoidance working together may be particularly disruptive.

A recent study indicated that daily assessments of experiential avoidance were a stronger predictor of daily well-being than the AAQ scores, measured daily. This indicates that mediational processes may be context specific [74]. Related to these measurement issues, there is conceptual confusion about the actual content measured with the AAQ-II that was suggested to measure acceptance and experiential avoidance, but also psychological flexibility [34]. Several measures derived from the AAQ have been developed to measure experiential avoidance or acceptance for different populations, such as people with diabetes or chronic pain [75]. Recently, the Multidimensional Experiential Avoidance Questionnaire was developed [76], focusing on several dimensions of experiential avoidance, e.g., behavioral avoidance and distress aversion, possibly allowing more fine-grained analyses. Further research should examine the relevance of the different dimensions in different contexts, such as different disorders, different levels of psychopathology and different situations. Of interest is whether experiential avoidance and its dimensions are stable and general coping factors, or whether they vary over time, per person and per situation.

### Strengths and limitations

The present study has a number of notable strengths. First, we compared two active conditions in treating a substantial number of patients with MDD in routine clinical practice. Second, the follow-up period of 12 months is longer than in many other trials. This is important as depression can be a perseverant disorder, with a high risk of relapse. Third, we made use of validated questionnaires to measure potential mediators. Other studies used measures specifically created for the study, which makes comparison with other studies difficult (e.g., [52, 55]). Fourth, our study meets four out of six of the criteria specified by Lemmens et al. [16] for testing mediation in psychotherapy research: the use of an randomized controlled trial design; inclusion of a control group; examination of multiple potential mediators within one study and the assessment of temporality. Participants in our study filled out the mediator and outcome measures at six different time points, during and after treatment. However, neither the criterion of direct experimental manipulation of the mediator, nor the criterion of required number of participants (*n* = 40) were met in this study. The CBT condition contained 38 participants which is 2 short to meet this criterion, and in both conditions we had missing data (note however that we used maximum likelihood estimation for the growth models to deal with missing data). Fifth, we used latent growth curve analysis and latent difference score modeling as up to date statistical approaches to establish mediation [19]. Candidate mediators were administered in both treatment conditions as previous research suggests that change in theorized processes may not be modality-specific (e.g., [77]), indicating that putative mediators may play a role in several, theoretically distinct treatments.

There are also several limitations to this study. The first limitation pertains to the required sample size to obtain sufficient statistical power to detect smaller effects, specifically concerning the mediational models in which treatment specific effects are tested. Although several effect size measures for mediation have been proposed, consensus has not been reached as to which, if any, of the existing effect size measures is recommended. Above, we have already discussed that our study just missed the sample size requirements as proposed by Lemmens et al. [16]. A more recent study by Pan et al. [78] provides specific guidelines to determine the required sample size in longitudinal mediation studies, accounting for within subject correlations across measurement occasions of the outcome. Based on the results of a simulation study they produce several tables that can be used to determine the required sample size to achieve 80% power, given the intra class correlation (ranging from .1 to .9) and the magnitude of the effect size (expressed as small, halfway, medium and large). Given the 6 measurement occasions of this study, and the intraclass correlation of .53 for the measure of depression (QIDS) used in this study, the tables of Pan et al. [78] suggest that our sample size was sufficient to detect halfway to medium effect sizes, whereas it was insufficient to detect small mediation effects, requiring sample sizes well over 200.

Second, looking at the absolute fit indices (i.e., RMSEA and CFI), model fit was poor for a substantive number of mediational models, in particular the latent difference score models, and all of the models in which decentering was included. This may imply that these models do not support the proposed mediational effects. Yet, the use of absolute fit indices such as RMSEA have recently become the target of sharp criticism as a number of simulation studies have demonstrated that absolute fit indices can lead to the false conclusion that a model fits acceptably when in fact the model is misspecified (e.g., [79]), which has led some authors to even suggest that these indices should no longer be used [80]. In addition, it is not uncommon to find that the absolute fit of a proposed model is poor, given the complexity of structural equation modeling [81], especially when considering models that aim to explain psychotherapeutic change. Variance of the measurement residuals leading to decreases in model fit can result from several factors [82], including random measurement error in the observed variable, occasion-specific nonsystematic variance, and occasion-specific systematic variance. Occasion-specific systematic variance is explainable by variables omitted from the model. In models that aim to explain psychotherapeutic change, omitted variables are inevitable as it will be impossible to incorporate all mechanisms of change, including specific effects (such as the underlying mechanisms that are the object of study in this manuscript), non-specific effects (such as warmth, respect, provision of a rationale), and the interplay of effects. As Lemmens et al. [16] conclude, even in the most optimal research designs, explaining psychotherapeutic change remains a challenge. We have mainly focused on BIC-values and the regression paths testing the hypothesized associations as, even when absolute fit indices indicate a poor fit, it is still theoretically meaningful when a) the BIC points to an improved fit of a model relative to an alternative model, and/or b) the mediational regression paths indicate significant associations.

Third, we did not include a waitlist control group. Especially long-term follow-up without a waitlist control-group risks overlooking all kinds of influences on changes in symptoms and quality of life. Life events, as well as personal strengths and vulnerabilities can influence outcomes in the long run. Yet, it is ethically and practically difficult to include a waitlist control group, or a placebo intervention for a long period of time while withholding patients proper treatment. Fourth, we used self-report measures of dysfunctional attitudes, decentering and experiential avoidance. Experience sampling methods or daily diary measurements (e.g., [68]) might be more suited to measure the fine-grained influence of these processes. This way, interactions of mediational processes can be measured, for instance by the use of cross-lagged panel designs [19]. We made a selection of mediators to investigate in this study. It is possible and even likely that other mediators may also have an effect. Both models are said to be stooled on several other processes, like behavioral activation, mindfulness, values and committed action. As mentioned above, the questionnaires we used in our study are not without concerns. On the other hand, the measures we applied are widely used in research, which makes comparison easier.

## Conclusions

Our findings support the long-term efficacy of both ACT and CBT. No significant differences in effect sizes were found between ACT and CBT. This suggests that both ACT and CBT are adequate treatments for depression. Outcomes were mediated by changes in dysfunctional attitudes, decentering and experiential avoidance. In addition, the results show that hypothesized treatment-specific processes did not mediate outcomes within the specified treatment only, with the exception of experiential avoidance, which specifically mediated outcome in the ACT condition. This suggests that experiential avoidance is specific to ACT and affirms this part of the ACT treatment model. Our results underscore earlier findings that cognitive change can be brought about in different ways, not limited to interventions from cognitive therapy. Our findings might inform further research on working mechanisms of both treatments for depression. Eventually, this research can help to improve the efficacy of depression treatments by informing us which processes we need to focus on during therapy and which related changes we need to facilitate. Because of the complexity of the subject, experimental studies with a clear, consistent theoretical framework are needed to further investigate meditators and mechanisms of change [72]. Until we have a clearer sense of how therapies work, ACT seems a viable alternative for CBT in the treatment of depression. However, replication studies on the efficacy of ACT are needed to gain the same level of confidence as the field has reached on the efficacy of CBT.

## Data Availability

The datasets used and/or analyzed during the current study are available from the corresponding author on reasonable request.

## Abbreviations

AAQ: Acceptance and Action Questionnaire
ACT: Acceptance and Commitment Therapy
BA: Behavioral Activation
BIC: Bayesian Information Criterion
CBT: Cognitive Behavioral Therapy
CFI: Comparative Fit Index
DAS: Dysfunctional Attitudes Scale
EQ-D: Decentering Scale of the Experiences Questionnaire
EUROHIS: European Health Interview Surveys Quality of Life Scale
MCAR: Missing Completely Ad Random
MDD: Major Depressive Disorder
LGCM: Latent Growth Curve Modeling
QIDS-SR: Quick Inventory of Depressive Symptomatology Self-Rated
RMSEA: Root Mean Square Error of Approximation
WHO: World Health Organization

## Footnotes

1 Although we report it in our tables, we have decided not to focus on the χ2-statistic as a measure of model fit, as it is no longer relied upon as a basis for goodness of fit of structural equation models, due to a number of disadvantages including the assumption of multivariate normality, and the lack of statistical power to discriminate between good fitting models and poor fitting models when smaller samples are used [45, 46]

## References

[1] Fact Sheet Depression [https://www.who.int/en/news-room/fact-sheets/detail/depression]. Accessed 13 Oct 2019.

[2] Hoppen TH, Morina N (2019). The prevalence of PTSD and major depression in the global population of adult war survivors: A meta-analytically informed estimate in absolute numbers. Eur J Psychotraumatol.

[3] Barth J, Munder T, Gerger H, Nüesch E, Trelle S, Znoj H, Jüni P, Cuijpers P (2013). Comparative efficacy of seven psychotherapeutic interventions for patients with depression: a network meta-analysis. PLoS Med.

[4] Cuijpers P, Gentili C (2017). Psychological treatments are as effective as pharmacotherapies in the treatment of adult depression: a summary from randomized clinical trials and neuroscience evidence. Research in Psychotherapy: Psychopathology, Process and Outcome.

[5] DeRubeis RJ, Cohen ZD, Forand NR, Fournier JC, Gelfand LA, Lorenzo-Luaces L (2014). The personalized advantage index: translating research on prediction into individualized treatment recommendations. A Demonstration. PLoS One.

[6] Bockting CL, Hollon SD, Jarrett RB, Kuyken W, Dobson KS (2015). A lifetime approach to major depressive disorder: the contributions of psychological interventions in preventing relapse and recurrence. Clin Psychol Rev.

[7] A-Tjak JGL, Morina N, Topper M, Emmelkamp PMG (2018). A randomized-controlled trial in routine clinical practice comparing acceptance and commitment therapy with cognitive behavioral therapy in the treatment of major depressive disorder. Psychother Psychosom.

[8] Folke F, Parling T, Melin L (2012). Acceptance and commitment therapy for depression: A preliminary randomized clinical trial for unemployed on long-term sick leave. Cognitive Behav Pract.

[9] Hayes L, Boyd CP, Sewell J (2011). Acceptance and commitment therapy for the treatment of adolescent depression: A pilot study in a psychiatric outpatient setting. Mindfulness.

[10] Kraemer HC, Wilson GT, Fairburn CG, Agras WS (2002). Mediators and moderators of treatment effects in randomized clinical trials. Arch Gen Psychiatry.

[11] Kazdin AE (2007). Mediators and mechanisms of change in psychotherapy research. Annu Rev Clin Psychol.

[12] Beck AT (1964). Thinking and depression II. Theory and therapy. Arch Gen Psychiatry.

[13] Hofmann SG, Admundson GJG, Beck AT (2013). The science of cognitive therapy. Behav Ther.

[14] Beck AT, Rush J, Shaw BF, Emery G (1979). Cognitive therapy of depression.

[15] Garratt G, Ingram RE, Rand KL, Sawalani G (2007). Cognitive processes in cognitive therapy: evaluation of the mechanisms of change in the treatment of depression. Clin Psychol.

[16] Lemmens LHJM, Müller VNLS, Arntz A, Huibers MJH (2016). Mechanisms of change in psychotherapy for depression: an empirical update and evaluation of research aimed at identifying psychological mediators. Clin Psychol Rev.

[17] Hayes SC, Strosahl KD, Wilson KG (2012). Acceptance and commitment therapy: the process and practice of mindful change second edition edn.

[18] Zettle RD, Rains JC, Hayes SC (2011). Processes of change in acceptance and commitment therapy and cognitive therapy for depression: A mediational reanalysis of Zettle and Rains (1989). Behav Modif.

[19] Selig JP, Preacher KJ (2009). Mediation models for longitudinal data in developmental research. Res Hum Dev.

[20] Forman EM, Chapman JE, Herbert JD, Goetter EM, Yuen EK, Moitra E (2012). Using session-by-session measurement to compare mechanisms of action for acceptance and commitment therapy and cognitive therapy. Behav Ther.

[21] Cuijpers P, Straten van A, Andersson G, Oppen van P (2008). Psychotherapy for depression in adults: A meta-analysis of comparative outcome studies. J Consult Clin Psychol.

[22] Tolin DF (2010). Is cognitive–behavioral therapy more effective than other therapies? A meta-analytic review. Clin Psychol Rev.

[23] First MB, Spitzer RL, Gibbon M, Williams JBW (1996). Structured clinical interview for DSM-IV Axis I disorders, clinician version (SCID-CV).

[24] Boelens W (2009). Behandelprotocol / Depressie / deel therapeutenboek + werkboek First edn.

[25] A-Tjak JGL (2015). Acceptance and Commitment Therapy. Theorie en Praktijk (Theory and Practice), Second edn.

[26] Zettle RD (2007). ACT for depression: A Clinician's guide to using Acceptance & Commitment Therapy in treating depression.

[27] Robinson P, Strosahl KD (2008). The mindfulness and acceptance workbook for depression: using acceptance and commitment therapy to move through depression and create a life worth living.

[28] Trivedi MH, Rush AJ, Ibrahim HM, Carmody TJ, Biggs MM, Suppes T, Crismon ML, Shores-Wilson K, Toprac MG, Dennehy EB (2004). The inventory of depressive symptomatology, clinician rating (IDS-C) and self-report (IDS-SR), the quick inventory of depressive Symtomatology, clinician rating (QIDS-C) and self-report (QIDS-SR) in public sector patients with mood disorders: a psychometric evaluation. Psychol Med.

[29] Hamilton M (1960). A rating scale for depression. J Neurol Neurosurg Psychiatry.

[30] Power MJ, Nosikov A, Gudex C (2003). Development of a common instrument for quality of life. Developing Common Instruments for Health Surveys.

[31] de Graaf LE, Roelofs J, Huibers MJH (2009). Measuring dysfunctional attitudes in the general population: the dysfunctional attitude scale (form A) revised. Cogn Ther Res.

[32] Beck AT (1987). Cognitive models of depression. J Cogn Psychother.

[33] Fresco DM, Moore MT, van Dulmen MH, Segal ZV, Ma SH, Teasdale JD, Williams JM (2007). Initial psychometric properties of the experiences questionnaire: validation of a self-report measure of decentering. Behav Ther.

[34] Bond FW, Hayes SC, Baer RA, Carpenter KC, Guenole N, Orcutt HK, Waltz T, Zettle RD (2011). Preliminary psychometric properties of the acceptance and action questionnaire – II: A revised measure of psychological flexibility and acceptance. Behav Ther.

[35] Jacobs N, Kleen M, De Groot F, A-Tjak JGL (2008). Het meten van experiëntiële vermijding. De Nederlandstalige versie van de acceptance and action questionnaire-II (AAQ II). [ the measurement of experiential avoidance. The Dutch language version of the acceptance and action questionnaire-II (AAQ-II)]. Gedragstherapie.

[36] Hox J (2010). Multilevel analysis techniques and applications, Second edn.

[37] Morris SB, DeShon RP (2002). Combining effect size estimates in meta-analysis with repeated measures and independent-groups designs. Psychol Methods.

[38] Morris SB (2008). Estimating effect sizes from pretest-posttest-control group designs. Organ Res Methods.

[39] Muthén LK, Muthén BO (2011). Mplus statistical modeling software (version 6.12).

[40] Bollen KA, Curran PJ (2006). Latent curve models: A structural equation perspective, vol. 467, First edn.

[41] Duncan TE, Duncan SC, Strycker LA (2013). An introduction to latent variable growth curve modeling: Concepts, issues, and application. , Second edn.

[42] Newsom JT (2015). Longitudinal structural equation modeling. A Comprehensive Introduction.

[43] Burnham KP, Anderson DR (2004). Multimodel inference: understanding AIC and BIC in model selection. Sociol Methods Res.

[44] Raftery A, Marsden P (1995). Bayesian model selection in social research. Sociological Methodology 1995 edn.

[45] Schermelleh-Engel K, Moosbrugger H, Müller H (2003). Evaluating the fit of structural equation models: tests of significance and descriptive goodness-of-fit measures. MPR-online.

[46] Kenny DA, McCoach DB (2003). Effect of the number of variables on measures of fit in structural equation modeling. Struct Equ Modeling.

[47] Hu LT, Bentler PM (1999). Cutoff criteria for fit indexes in covariance structure analysis: conventional criteria versus new alternatives. Struct Equ Modeling.

[48] MacKinnon DP (2008). Multivariate applications series. Introduction to statistical mediation analysis.

[49] McArdle JJ, Hamagami F, Collins LM, Sayer AG (2001). Latent difference score structural models for linear dynamic analyses with incomplete longitudinal data. Decade of behavior New methods for the analysis of change.

[50] A-Tjak JGL, Davis ML, Morina N, Powers MB, Smits JA, Emmelkamp PMG (2015). A meta-analysis of the efficacy of acceptance and commitment therapy for clinically relevant mental and physical health problems. Psychother Psychosom.

[51] Ost LG (2014). The efficacy of acceptance and commitment therapy: an updated systematic review and meta-analysis. Behav Res Ther.

[52] Forman EM, Shaw JA, Goetter EM, Herbert JD, Park JA, Yuen EK (2012). Long-term follow-up of a randomized controlled trial comparing acceptance and commitment therapy and standard cognitive behavior therapy for anxiety and depression. Behav Ther.

[53] Barlow DH, Sauer-Zavala S, Carl JR, Bullis JR, Ellard KK (2014). The nature, diagnosis, and treatment of neuroticism: Back to the future. Clin Psychol Sci.

[54] Cristea IA, Huibers MJH, David D, Hollon SD, Andersson G, Cuijpers P (2015). The effects of cognitive behavior therapy for adult depression on dysfunctional thinking: A meta-analysis. Clin Psychol Rev.

[55] van Luenen S, Kraaij V, Spinhoven P, Wilderjans TF, Garnefski N (2019). Exploring mediators of a guided web-based self-help intervention for people with HIV and depressive symptoms: randomized controlled trial. JMIR Ment Health.

[56] Segal ZV, Williams JMG, Teasdale JD (2002). Mindfulness-based cognitive therapy for depression: A new approach to preventing relapse.

[57] Bieling PJ, Hawley LL, Bloch RT, Corcoran KM, Levitan RD, Young LT, Macqueen GM, Segal ZV (2012). Treatment-specific changes in decentering following mindfulness-based cognitive therapy versus antidepressant medication or placebo for prevention of depressive relapse. J Consult Clin Psychol.

[58] Fresco DM, Segal ZV, Buis T, Kennedy S (2007). Relationship of Posttreatment decentering and cognitive reactivity to relapse in major depression. J Consult Clin Psychol.

[59] Lo CSL, Ho SMY, Yu NKK, Siu BPY (2014). Decentering mediates the effect of ruminative and experiential self-focus on negative thinking in depression. Cogn Ther Res.

[60] McCracken LM, Barker E, Chilcot J (2014). Decentering, rumination, cognitive defusion, and psychological flexibility in people with chronic pain. J Behav Med.

[61] Dimidjian S, Arch JJ, Schneider R, Desormeau P, Felder JN, Segal ZV (2016). Considering meta-analysis, meaning, and metaphor: A systematic review and critical examination of “third wave” cognitive and behavioral therapies. Behav Ther.

[62] Zettle RD, Hayes SC (1986). Dysfunctional control by client verbal behavior: the context of reason giving. Anal Verbal Behav.

[63] Zettle RD, Rains JC (1989). Group cognitive and contextual therapies in treatment of depression. J Clin Psychol.

[64] Gecht J, Kessel R, Forkmann T, Gauggel S, Drueke B, Scherer A, Mainz V (2014). A mediation model of mindfulness and decentering: sequential psychological constructs or one and the same?. BMC Psychol.

[65] Gillanders DT, Bolderston H, Bond FW, Dempster M, Flaxman PE, Campbell L, Kerr S, Tansey L, Noel P, Ferenbach C (2014). The development and initial validation of the cognitive fusion questionnaire. Behav Ther.

[66] Levin ME, Luoma JB, Haeger JA (2015). Decoupling as a mechanism of change in mindfulness and acceptance: A literature review. Behav Modif.

[67] Cribb G, Moulds ML, Carter S (2006). Rumination and experiential avoidance in depression. Behav Change.

[68] Shahar B, Herr NR (2011). Depressive symptoms predict inflexibly high levels of experiential avoidance in response to daily negative affect: A daily diary study. Behav Res Ther.

[69] Fledderus M, Bohlmeijer ET, Fox GJA, Schreurs KMG, Spinhoven P (2013). The role of psychological flexibility in a self-help acceptance and commitment therapy intervention for psychological distress in a randomized controlled trial. Behav Res Ther.

[70] Bohlmeijer ET, Lamers SMA, Fledderus M (2015). Flourishing in people with depressive symptomatology increases with acceptance and commitment therapy. Post-hoc analyses of a randomized controlled trial. Behav Res Ther.

[71] Van der Zanden R, Galindo-Garre F, Curie K, Kramer J, Cuijpers P (2014). Online cognitive-based intervention for depression: exploring possible circularity in mechanisms of change. Psychol Med.

[72] Cuijpers P, Reijnders M, Huibers MJH (2019). The role of common factors in psychotherapy outcomes. Rev Clin Psychol.

[73] Bardeen JR, Fergus TA (2016). The interactive effect of cognitive fusion and experiential avoidance on anxiety, depression, stress and posttraumatic stress symptoms. J Contextual Behav Sci.

[74] Machell KA, Goodman FR, Kashdan TB (2015). Experiential avoidance and well-being: A daily diary analysis. Cognit Emot.

[75] Batink T, Jansen G, Peeters FPML (2015). Nieuwe generatie gedragstherapie, nieuwe generatie meetinstrumenten. Tijdschr Psychiatr.

[76] Gámez W, Chmielewski M, Kotov R, Ruggero C, Watson D (2011). Development of a measure of experiential avoidance: the multidimensional experiential avoidance questionnaire. Psychol Assess.

[77] Warmerdam L, van Straten A, Jongsma J, Twisk J, Cuijpers P (2010). Online cognitive behavioral therapy and problem-solving therapy for depressive symptoms: exploring mechanisms of change. J Behav Ther Exp Psychiatry.

[78] Pan H, Liu S, Miao D, Yuan Y. Sample size determination for mediation analysis of longitudinal data. BMC Med Res Methodol. 2018;18(1):32. 10.1186/s12874-018-0473-2 PMC5870539 29580203

[79] Bauducel A, Wittmann W (2005). Simulation study on fit indices in confirmatory factor analysis based on data with slightly distorted simple structure. Struct Equ Modeling.

[80] Barrett B (2007). Structural equation modelling: adjudging model fit. Pers Individ Dif.

[81] Hooper D, Coughlan J, Mullen M (2008). Evaluating model fit: a synthesis of the structural equation modelling literature. 7th European Conference on research methodology for business and management studies.

[82] Bollen KA, Cudeck R, MacCallum R (2007). On the origins of latent curve models. Factor analysis at 100.

